# Hepatitis C Virus Induces the Cannabinoid Receptor 1

**DOI:** 10.1371/journal.pone.0012841

**Published:** 2010-09-17

**Authors:** David van der Poorten, Mahsa Shahidi, Enoch Tay, Jayshree Sesha, Kayla Tran, Duncan McLeod, Jane S. Milliken, Vikki Ho, Lionel W. Hebbard, Mark W. Douglas, Jacob George

**Affiliations:** 1 Storr Liver Unit, Westmead Millennium Institute, University of Sydney at Westmead Hospital, Sydney, Australia; 2 Department of Anatomical Pathology, Institute of Clinical Pathology and Medical Research (ICPMR), Westmead Hospital, Sydney, Australia; 3 Centre for Infectious Diseases and Microbiology, Westmead Hospital, Sydney, Australia; Institut Pasteur, France

## Abstract

**Background:**

Activation of hepatic CB_1_ receptors (CB_1_) is associated with steatosis and fibrosis in experimental forms of liver disease. However, CB_1_ expression has not been assessed in patients with chronic hepatitis C (CHC), a disease associated with insulin resistance, steatosis and metabolic disturbance. We aimed to determine the importance and explore the associations of CB_1_ expression in CHC.

**Methods:**

CB_1_ receptor mRNA was measured by real time quantitative PCR on extracted liver tissue from 88 patients with CHC (genotypes 1 and 3), 12 controls and 10 patients with chronic hepatitis B (CHB). The Huh7/JFH1 Hepatitis C virus (HCV) cell culture model was used to validate results.

**Principal Findings:**

CB_1_ was expressed in all patients with CHC and levels were 6-fold higher than in controls (*P*<0.001). CB_1_ expression increased with fibrosis stage, with cirrhotics having up to a 2 fold up-regulation compared to those with low fibrosis stage (*p*<0.05). Even in mild CHC with no steatosis (F0-1), CB_1_ levels remained substantially greater than in controls (*p*<0.001) and in those with mild CHB (F0-1; *p*<0.001). Huh7 cells infected with JFH-1 HCV showed an 8-fold upregulation of CB_1_, and CB_1_ expression directly correlated with the percentage of cells infected over time, suggesting that CB_1_ is an HCV inducible gene. While HCV structural proteins appear essential for CB_1_ induction, there was no core genotype specific difference in CB_1_ expression. CB_1_ significantly increased with steatosis grade, primarily driven by patients with genotype 3 CHC. In genotype 3 patients, CB_1_ correlated with SREBP-1c and its downstream target FASN (SREBP-1c; R = 0.37, FASN; R = 0.39, p<0.05 for both).

**Conclusions/Significance:**

CB_1_ is up-regulated in CHC and is associated with increased steatosis in genotype 3. It is induced by the hepatitis C virus.

## Introduction

Chronic Hepatitis C (CHC) is one of the most common causes of hepatic fibrosis and cirrhosis with the World Health Organization (WHO) estimating that up to 3% (180 million people) of the world’s population are affected [1]. The pathogenic processes by which hepatitis C virus (HCV) causes liver fibrosis are incompletely understood, but include immune activation, direct cytopathic effects, activation of hepatic stellate cells, induction of insulin resistance and hepatic steatosis [2]. A number of clinical factors are associated with fibrosis progression in CHC including male gender, duration of infection, age at infection, excessive alcohol use [3] and most recently, daily cannabis smoking [4]. There are genotype-specific associations with steatosis: HCV genotype 1 induces steatosis in association with insulin resistance [5]; HCV genotype 3 directly induces steatosis [6] independent of other metabolic risk factors, which resolves following successful anti-viral therapy [7]. Steatosis in CHC is associated with liver fibrosis [8], an increased risk of liver cancer [9], and higher levels of viral replication [10].

Cannabis (*Cannabis Sativa*, marijuana) has been used for medicinal and ritual purposes for over 3 millennia, and remains the most commonly used recreational drug in the western world [11]. The identification of the cannabinoid receptor 1 (CB_1_) in human brain some twenty years ago [12] and the subsequent discovery of endogenous cannabinoids, has led to an understanding of the importance of the endocannabinoid system in health and disease. There are two G protein-coupled cannabinoid receptors; CB_1_ and CB_2_ [13]. CB_1_ is found in high concentrations in the brain, but is also present in many peripheral tissues such as the liver, adipose tissue and gut. CB_2_ is found primarily in the immune system, but is also expressed in peripheral tissues including the liver [14]. The two best characterised endocannabinoids (ECBs) are arachidonoylethanolamide (anandamide) and 2-arachidonoyl-glycerol (2-AG). ECBs acting through CB_1_ have a strong anabolic effect and play an important role in appetite stimulation and normal energy homeostasis [14], [15]. CB_1_ blockade confers resistance to the development of diet-induced obesity [16], increases adiponectin levels, reduces triglyceride levels and causes weight loss independent of food intake [17], [18]. CB_1_ activation therefore is associated with obesity, insulin resistance and dyslipidaemia.

Data detailing the importance of the endocannabinoid system to hepatic disease remains limited. CB_1_ and CB_2_ receptors are weakly expressed in normal liver, but are strongly up-regulated in experimental liver injury and cirrhosis due to alcohol, hepatitis B, and primary biliary cirrhosis [19]. CB_1_ inactivation has been shown to inhibit the progression of fibrosis in three models of liver injury [19]; conversely, CB_2_ blockade enhances experimental liver fibrosis [20] and CB_2_ activation causes partial fibrotic reversal in cirrhotic rats [21]. CB_1_ receptors have been shown to mediate both alcohol [22] and diet [18] induced hepatic steatosis by up regulating the lipogenic transcription factor SREBP-1c and increasing *de novo* fatty acid synthesis [18]. In the obese Zucker rat model, treatment with a CB_1_ antagonist abolished hepatomegaly and steatosis, and caused normalisation of liver enzymes [23]. Furthermore, in well characterised cohorts with CHC, daily cannabis use, which was reported in 32% of patients, was significantly associated with the progression of fibrosis and the development of severe steatosis and fibrosis [4], [24].

Thus, there is good reason to believe that the endocannabinoid system is of importance in hepatitis C and may be involved in the metabolic dysregulation, hepatic steatosis, hepatic fibrogenesis and insulin resistance of CHC. To date however, the cannabinoid receptors have not been definitively identified in association with hepatitis C, nor has their significance been directly examined. In this study we demonstrate for the first time that the CB_1_ receptor is present in all patients with hepatitis C and is significantly up-regulated when compared with controls. We show that CB_1_ receptor expression increases with fibrosis stage and is associated with increased steatosis. Moreover, through the use of the Huh7/JFH1 HCV cell culture model, we demonstrate that CB_1_ up-regulation is also a viral effect, independent of hepatic inflammation and fibrosis.

## Methods

### Ethics statement

The study protocol was approved by the Human Ethics Committee of the Western Sydney Area Health Service and written informed consent was obtained from all participants.

### Patient selection

Study subjects were selected from a prospectively collected database of over 400 patients with chronic HCV infection who underwent liver biopsy at Westmead Hospital. All subjects had antibodies against HCV (Monolisa anti-HCV; Sanofi Diagnostics Pasteur, Marnes-la-Coquette, France) and detectable HCV RNA by PCR (Amplicor HCV; Roche Diagnostics, Branchburg, NJ, USA). Hepatitis C virus genotyping was performed with a second generation reverse hybridization line probe assay (Inno-Lipa HCV II; Innogenetics, Zwijndrecht, Belgium). Of 446 patients in total, only the 372 with genotype 1 or 3 disease were included. Of these, 193 patients with additional risk factors for liver steatosis or fibrosis other than HCV; ie those with diabetes, obesity (BMI>30 kg/m^2^), significant alcohol intake (>20 g/day) or dyslipidaemia (Total cholesterol >5.5 mmol/L, LDL >4 mmol/L, HDL <1 mmol/L or TG >2 mmol/L) were excluded. 87 were excluded due to lack of stored liver tissue or serum, or poor quality RNA. 11% of the cohort had smoked cannabis within the last year. Four patients who used cannabis daily were excluded in line with recent data showing that only regular daily use is a risk factor for the progression of fibrosis and steatosis [4], [24]. This left 88 study participants. No patient had clinical evidence of hepatic decompensation at the time of biopsy.

### Clinical and Laboratory Evaluation

A complete physical examination was performed on each subject. On the morning of the liver biopsy, venous blood was drawn after a 12 hour overnight fast to determine the serum levels of alanine aminotransferase (ALT), albumin, bilirubin, platelet count, international normalized ratio, glucose and insulin. Hepatitis C viral load was measured by PCR (Amplicor HCV; Roche Diagnostics, Branchburg, NJ, USA) with a dynamic range of 100–850,000 IU/mL. Serum insulin was determined by radio-immunoassay (Phadaseph insulin RIA; Pharmacia and Upjohn Diagnostics AB, Uppsala, Sweden). Insulin resistance was calculated by the homeostasis model (HOMA-IR) using the following formula: HOMA-IR  =  fasting insulin (mU/L) × plasma glucose (mmol/L)/22.5. All other biochemical tests were performed using a conventional automated analyzer within the Department of Clinical Chemistry at Westmead Hospital.

### Histopathology

All liver biopsy specimens were scored semi-quantitatively using the Scheuer score [25] by an experienced hepatopathologist blinded to clinical data. Portal/periportal inflammatory grade and fibrosis stage was scored from 0 to 4. Steatosis was graded 0 to 3 as follows; 0: <2% fat, 1: 2–10% fat, 2: 10–30% fat, 3: >30% fat. Patients with steatosis grades 2–3 were grouped together for statistical purposes.

### Control and Hepatitis B subjects

Twelve healthy controls had a core liver biopsy at the time of cholecystectomy or benign tumor resection. All had normal liver tests, negative serology for chronic viral hepatitis and no history of liver disease or T2DM and normal liver histology. Ten patients with chronic hepatitis B, low fibrosis and no steatosis on biopsy (F0-1) were selected from a prospectively collected database. These patients had a positive HBsAg, and raised ALT at the time of biopsy.

### Huh7/JFH-1 (Japanese fulminant hepatitis) cell line

Huh7 cells were transfected with JFH-1 strain of hepatitis C by electroporation and passaged in culture for 3 weeks until over 90% of cells were infected, as previously described [26]. HCV infection was confirmed by immunofluorescence, using antibodies against HCV NS5A protein (provided by Prof Mark Harris, University of Leeds). For the time course studies, Huh7 cells were infected by incubating overnight with supernatant from JFH-1 infected Huh7 cells. Cells were then monitored for 26 days, with HCV infection confirmed by immunofluorescence microscopy as above.

### Subgenomic replicon and Genotype 1 and 3 Chimeric virus

Huh7 cells were transfected with a subgenomic replicon based on the JFH-1 HCV strain, expressing nonstructural proteins NS3 to NS5B and containing a neomycin (G418) resistance gene [27]. Cells were passaged for 3 weeks in G418 (250 µg/mL) until only transfected cells survived. Immunofluorescence confirmed that over 90% of cells were infected. Chimeric viruses containing core protein from genotype 1b (N strain) or genotype 3a (HCV3a-GLa) [28] were used to transfect Huh7 cells as described above. Cells were passaged in culture until over 90% were infected.

### RNA extraction and cDNA synthesis

Total RNA was isolated from liver and cell culture samples using the RNeasy Mini Kit (Qiagen, Hilden, Germany) according to the manufacturer's protocol. RNA quality analysis was then performed using an Agilent 2100 Bioanalyser (Agilent Technologies, Palo Alto, CA, USA) as per the manufacturer's instructions. Total RNA with an integrity number >7 was considered acceptable. 200 ng and 1 ug of liver and cell RNA respectively was then reverse transcribed to first strand complementary DNA (cDNA) using Superscript III RT kit (Invitrogen, Carlsbad, CA, USA) and random primers.

### Gene expression by Real-time PCR

Real-time quantitative PCR (qPCR) was performed using a Corbett Rotor-gene 6000 (Corbett life sciences, Mortlake, Australia). Amplifications were performed in a 10 uL reaction containing 4 uL of cDNA, 5 uL of Platinum qPCR Super-mix (Invitrogen, Carlsbad, CA, USA) and 0.25 uL of either CB_1_, SREBP-1c or FASN Taqman primer probe (Applied Biosystems, Foster City, CA, USA). Amplification conditions were according to the manufacturer's protocol. The housekeeper gene 18S was used as an internal control. CB_1_ mRNA was quantitated using Corbett Rotor-gene series software v1.7 (Corbett life sciences, Mortlake, Australia) and values were expressed relative to 18S. For all cell culture experiments, 3 replicates of control and infected cells were assayed and the mean values reported.

### Western Blot and Immunohistochemistry

The relative tissue content of CB_1_ protein was assessed by western blot analysis using CB_1_ receptor antibody (Sigma, product no. C1233). Cells or liver biopsy tissue was processed using the Proteo-extract ™ sub cellular proteome extraction kit (Calbiochem, San Diego, USA) to purify membrane fraction associated protein. Protein (100 µg) was run on a 10% PAGE gel and blotted onto nitrocellulose membranes. Membranes were blocked with 5% skim milk powder in TBST (0.1% Tween) for 1 hour and incubated overnight at 4°C with anti-CB_1_ antibody at a dilution of 1:1000 (diluted in 5% skim milk powder/TBST). Membrane were then washed 3X in TBST and incubated with appropriate horse-radish peroxidase conjugated secondary antibody and the resulting signal detected using the Supersignal luminescent detection system (Thermo Scientific, Rockford IL, USA). CB_1_ bands were further quantitated by densitometry using Image J software (ImageJ, NIH, Bethesda USA [29]), with values normalised to the loading control dye (Amido Black). For immunohistochemistry, formalin fixed, paraffin embedded 4 µsections were stained using a Ventana Benchmark Immunostainer (Ventana Medical Systems, Inc, Arizona, USA). Detection was performed using Ventana's Ultra View DAB kit (Roche/Ventana 05269806001) using the following protocol: The sections were dewaxed with Ventana EZ Prep. Endogenous peroxidase activity was blocked using the Ventana inhibitor in the kit. Anti cannabinoid receptor 1 antibody (Cayman, product no. 10006590; Cayman Chemical, Ann Arbor, MI, USA) was diluted in Biocare's DaVinci Green diluent (Biocare Medical-Concord, CA 94520) for 32 mins at 42°C. The site of the antigen was visualised with Ventana's Ultra View DAB kit. The sections were counterstained with Ventana Haematoxylin and blued with Ventana Blueing Solution. On completion of staining the sections were dehydrated in alcohol, cleared in Xylene and mounted in Permount. Negative controls where the primary antibody was excluded confirmed the specificity of immunostaining.

### Statistical Analysis

Statistical analysis was carried out using SPSS version 16.0 (SPSS Inc., Chicago, IL). Results are reported as mean ± standard deviation (SD). Univariate analysis of variance (ANOVA) was used to examine factors associated with increasing histology grades/stages as these were categorical variables with multiple end-points. Student *t*-tests were used to compare means of continuous variables. The strength of association between continuous variables was reported using Spearman rank correlations due to the non-parametric nature of certain variables. Multiple ordinal regression analysis was performed to determine the independent associations of viral load, steatosis grade and fibrosis stage. For the steatosis and fibrosis models all variables significant on univariate analysis were entered, and backward stepwise removal of variables to create a best-fitting model was performed. An interaction term (genotype multiplied by CB_1_) was used in the steatosis model to determine if the association between CB_1_ and steatosis was genotype dependent. P-values of <0.05 were considered significant.

## Results

The baseline characteristics of the 88 patients with chronic hepatitis C is presented in table 1. The mean age for these patients was 42, with the majority male (64.8%) and of normal body mass. 56% had genotype 1 disease and 44% had genotype 3 infection. Over a third had advanced fibrosis (F3–4; 37.5%) and steatosis was present in 54.5%. Control patients are compared to the 31 hepatitis C patients with low fibrosis (F0-1) and no steatosis, and to 10 patients with chronic hepatitis B in table 2. Controls had a similar mean age to those with hepatitis C, but were more insulin resistant, obese and contained a lower percentage of males. Control liver biopsies were histologically normal. The 10 hepatitis B patients studied all had low fibrosis (F0-1), but comparable hepatic inflammation to those with hepatitis C.

**Table 1:** Baseline characteristics of patients with Chronic hepatitis C.

	Hepatitis C
	(n = 88)
Age	42.6 (9.7)
Sex (male)	57 (64.8%)
BMI	24.9 (2.9)
Genotype 1	49 (56%)
Genotype 3	39 (44%)
Fibrosis Stage	
0	12 (13.6%)
1	39 (44.3%)
2	4 (4.5%)
3	20 (22.7%)
4	13 (14.8%)
Steatosis Grade	
0	40 (45.5%)
1	22 (25%)
2	22 (25%)
3	4 (4.5%)
Portal Inflammation Grade	
1	11 (12.5%)
2	39 (44.3%)
3	22 (25%)

Variables are reported as mean (SD) or frequency (percentage) as appropriate.

**Table 2:** Baseline characteristics of patients with Chronic Hepatitis C (F0-1), Chronic hepatitis B (F0-1) and controls.

	Hepatitis C (F0-1)	Hepatitis B (F0-1)	P-value*	Control	P-value**
	(*n* = 31)	(*n* = 10)		(*n* = 12)	
Age	39.7 (11.1)	37 (11.8)	0.44	42.2 (9.4)	0.5
Sex (male)	16 (51.%)	8 (80%)	0.3	3 (25%)	<0.01
BMI	24.1 (2.6)	22.7 (2.9)	0.1	29.6 (9.8)	<0.01
HOMA-IR	1.7 (0.9)	1.4 (1.3)	0.5	2.4 (1.1)	0.04
Fibrosis Stage					
0–1	31 (100%)	10 (100%)	-	12 (100%)	-
2–4	0	0		0	
Steatosis Grade					
0	31 (100%)	10 (100%)	-	12 (100%)	-
1–3	0	0		0	
Portal Inflammation Grade					
1	7 (22.6%)	4 (40%)	0.4	0	-
2–3	24 (77.4%)	6 (60%)		0	

Variables are reported as mean (SD) or frequency (percentage) as appropriate.

*p-values for Hepatitis C (F0-1) and Hepatitis B (F0-1), **p-values for Hepatitis C (F0-1) and control.

### CB_1_ expression in hepatitis C, controls and hepatitis B

**Figure 1:**
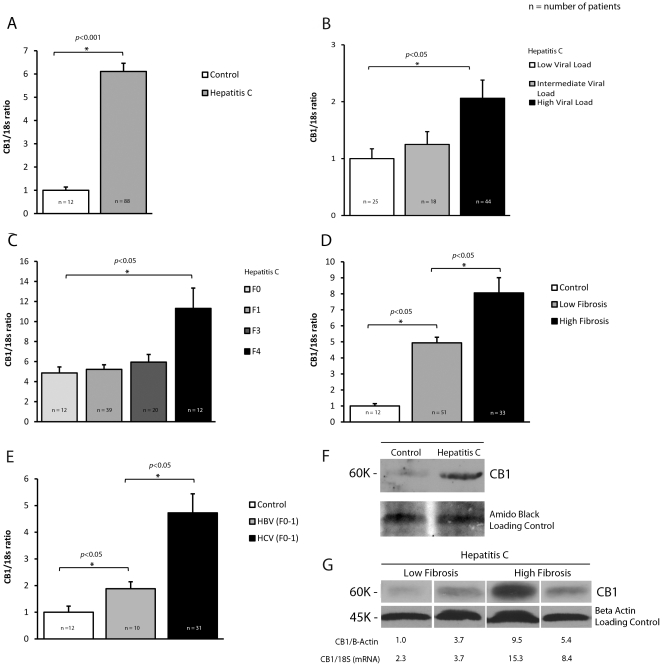
CB_1_ is up-regulated in chronic hepatitis C and associated with viral load and fibrosis. Relative hepatic CB_1_ mRNA expression in 88 patients with Chronic Hepatitis C, 12 controls and 10 patients with chronic hepatitis B normalised to 18s;, A) 6-fold up-regulation of CB_1_ in hepatitis C patients compared to control, B) Viral load: Significant association between CB_1_ expression and increasing viral load. C) Fibrosis stage: Increasing expression of CB_1_ with increasing fibrosis stage in hepatitis C, D) Significant up-regulation in CB_1_ even in those with low fibrosis stage and no steatosis (F0-1) compared to control, E) Hepatitis B patients with low fibrosis had increased CB_1_ expression when compared to control, but at significantly lower levels than hepatitis C patients with low fibrosis, F) Western blot from representative control and hepatitis C patients demonstrating significant CB_1_ up-regulation and G) Western blot from representative patients with hepatitis C and differing levels of fibrosis, showing increased CB_1_ expression in patients with high fibrosis. The relative protein expression (CB_1_/B-Actin) and mRNA expression (CB_1_/18S) are presented for validation.

CB_1_ was expressed in all patients with hepatitis C, and there was a 6-fold up-regulation when compared to controls (*P*<0.001, figure 1A and 1F). Within the hepatitis C cohort, CB_1_ expression significantly correlated with increasing viral load (figure 1B). Patients with a high viral load (>800,000 IU/ml) had significantly higher CB_1_ than those with intermediate (400,000–800,000 IU/mL), or low viral load (<400,000 IU/mL, *p* = 0.03), even when controlled for fibrosis stage (*p* = 0.05). There was no difference in CB_1_ expression between those who had smoked cannabis in the last year (n = 10) and those who had not.

CB_1_ expression increased with increasing fibrosis stage, with cirrhotics having up to a 2 fold up-regulation compared to those with low fibrosis stage (F0/1- figure 1C) and results were confirmed on tissue lysates by western blot (figure 1G). Despite this relationship to fibrosis, CB_1_ levels in hepatitis C patients with low fibrosis and no steatosis were still substantially greater than those in controls (*p*<0.05, figure 1D).

To determine if CB_1_ gene expression was a non-specific response to virus-mediated liver injury, we next compared CB_1_ expression in 10 patients with hepatitis B and low fibrosis to the controls and to hepatitis C patients with low fibrosis and no steatosis. In the hepatitis B patients, CB_1_ expression was increased when compared with controls, but was almost three-fold lower than that seen in a similar cohort with hepatitis C (figure 1E).

### JFH-1/Huh7 HCV cell culture model and genotype 1 and 3 chimeric virus

**Figure 2:**
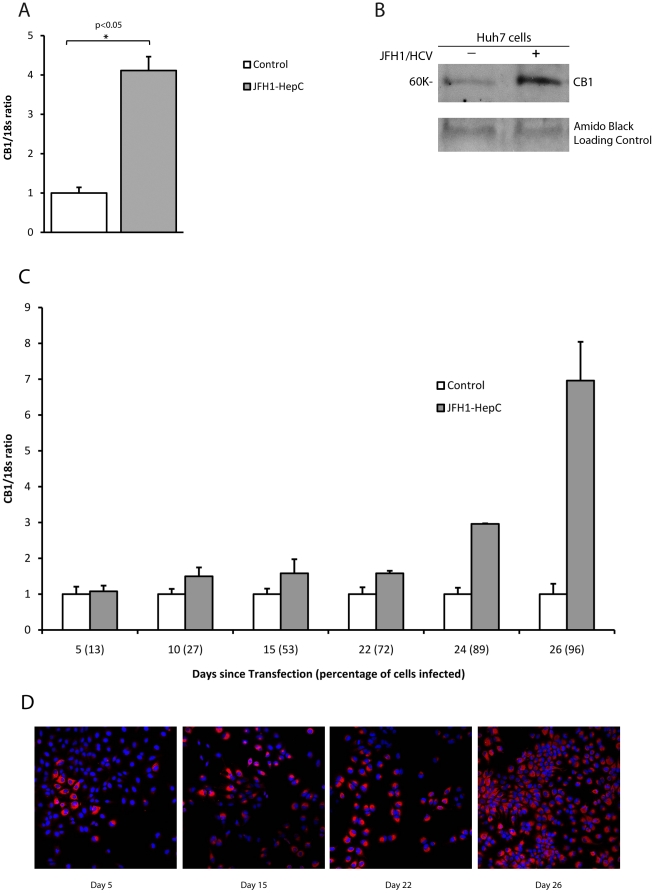
CB_1_ is directly up-regulated by hepatitis C virus in a cell culture system. Relative hepatic CB_1_ mRNA expression in Huh7 cells infected with the JFH_1_ strain hepatitis C virus as compared to mock infected control cells normalised to 18s; A) 4-fold up-regulation of CB_1_ mRNA in JFH-1 infected cells compared to control and B) Western blotting confirmed CB_1_ protein up-regulation by at least >8-fold as measured by densitometry. C) Time course of CB_1_ expression following de novo infection with JFH-1 hepatitis C virus. CB_1_ expression increases with time (*p*<0.01), in parallel to the percentage of Huh7 cells infected (see horizontal axis) D) representative immunostaining for NS5a showing increasing infection of Huh7 cells at day 5, 15, 22 and 26.

To exclude any potential changes that could be due to fibrosis or the injury milieu in the liver and to determine if CB_1_ up-regulation is in part a HCV-specific effect, we assessed receptor expression in the JFH-1/Huh7 model of replicating virus *in vitro*. Huh7 cells infected with the JFH1 strain of HCV showed a 4-fold upregulation of CB_1_ mRNA compared to control Huh7 cells (figure 2A, *p*<0.05). Immunoblotting confirmed an 8-fold induction of CB_1_ protein as measured by densitometry (figure 2B). We next examined the expression of CB_1_ over time following *de novo* infection of Huh7 cells with JFH-1. CB_1_ expression increased slowly between day 5-22 and then rapidly between day 22–26 (p <0.001 for change in CB_1_, figure 2C) Importantly, the changes in CB_1_ expression paralleled increasing HCV infection, in particular when over 50% of cells were infected (R = 0.73, figure 2C and D).

**Figure 3:**
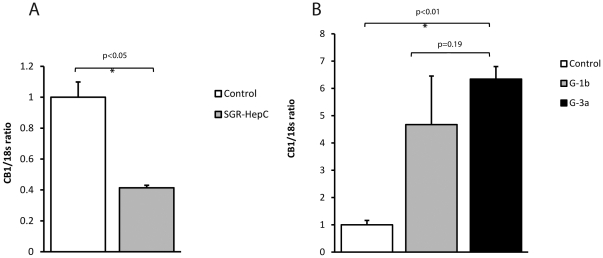
CB_1_ expression in a subgenomic HCV replicon and genotype specific chimeric virus. Relative hepatic CB_1_ mRNA expression in Huh7 cells infected either with a subgenomic HCV replicon (expressing JFH-1 NS3-NS5B) or genotype specific chimeric virus as compared to control. A) There was a 60% reduction in CB_1_ expression in the HCV replicon containing cells compared to control and B) There was a 4.7 and 6.3 fold up-regulation in CB_1_ expression in the genotype 1b and 3a chimera viruses when compared to control. There was no difference, however, in CB_1_ expression between the two different genotype chimeras.

To determine if CB_1_ induction was due to structural or non-structural viral proteins, we next transfected Huh7 cells with a subgenomic replicon expressing only the non-structural proteins NS3 to NS5B. Compared with control, there was a 60% reduction in CB_1_ expression in the HCV replicon containing cells (figure 3A), suggesting that HCV structural proteins are essential for promoting CB_1_ expression in HCV infection.

We therefore went on to investigate the genotype-specific effect of HCV structural proteins on CB_1_ expression using chimeric viruses containing core protein from genotype 1b and genotype 3a. CB_1_ expression in Huh7 cells infected with chimeric HCV increased as the proportion of infected cells increased. This was similar to the results obtained using wild type JFH-1 (data not shown). When over 90% of the cells were infected, there was a corresponding 4.7 and 6.3 fold up-regulation of CB_1_ from genotype 1b and 3a chimera's respectively, as compared to control Huh7 cells (p<0.01, figure 3B). However, there was no difference in the up-regulation of CB_1_ between genotypes 1b and 3a (*p* = 0.19), suggesting that although the HCV structural proteins are essential for CB_1_ induction, there is no genotype-specific effect of core protein.

### Immunohistochemistry in Hepatitis C

**Figure 4:**
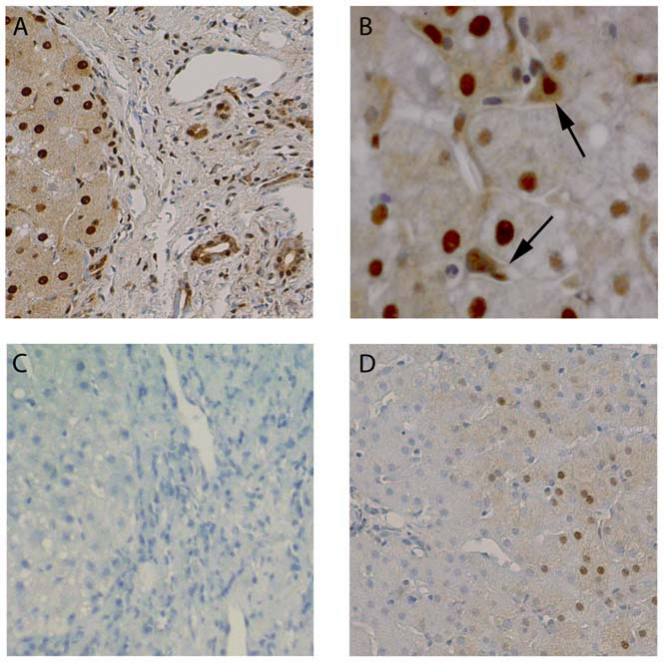
Representative immunostaining for CB_1_ receptor protein in hepatitis C patients with high and low CB_1_ expression. High CB_1_ expression and advanced fibrosis: A) strong, diffuse cytoplasmic and nuclear immunostaining of hepatocytes is evident in addition to cholangiocyte and B) hepatic stellate cell immunostaining (arrows). Negative control: C) No immunostaining apparent in negative control where the primary antibody was excluded. Low CB_1_ expression and low fibrosis: D) low intensity and patchy cytoplasmic and nuclear immunostaining of hepatocytes is evident.

CB_1_ receptor protein expression by immunohistochemistry correlated with RNA expression by qPCR. Patients with high CB_1_ expression exhibited diffuse cytoplasmic and nuclear staining of hepatocytes in addition to strong staining of hepatic stellate cells and cholangiocytes (figure 4A and B). Immunostaining in patients with low CB_1_ expression and low fibrosis was less intense, patchy and confined to hepatocytes (figure 4D). A negative control image where the primary antibody was excluded is provided (figure 4C) to demonstrate the specificity of immunostaining. Low power images in patients with high and low fibrosis respectively are presented as supplementary material (Figure S1). The nuclear localisation of CB_1_ receptors is in keeping with recent evidence that trans-membrane G-protein coupled receptors can internalise on the cell nucleus [30], [31].

### The relationship of CB_1_ expression to hepatic inflammation and steatosis

**Figure 5:**
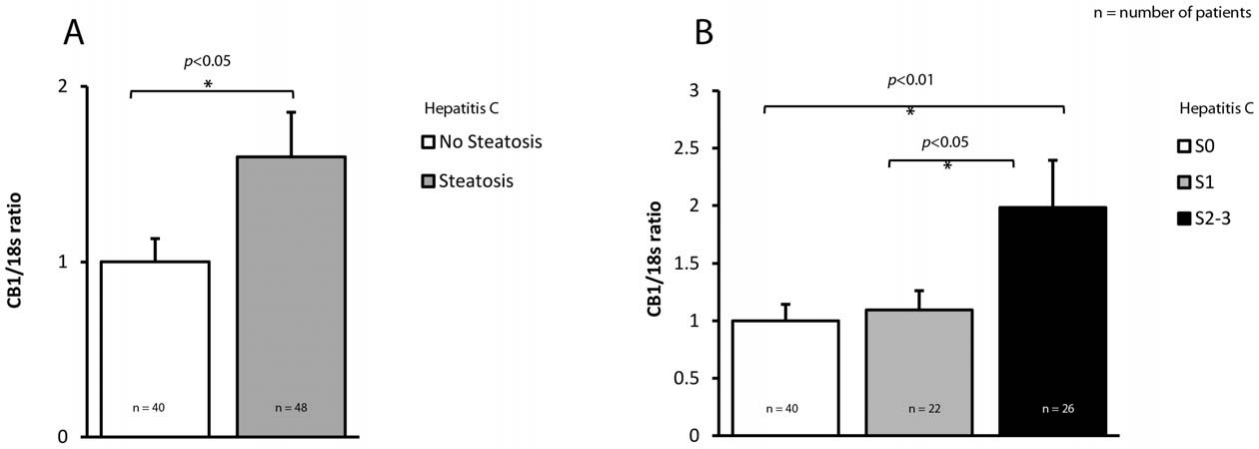
CB_1_ expression is associated with increasing steatosis in chronic hepatitis C. Relative CB_1_ mRNA expression in 88 patients with Chronic Hepatitis C normalised to 18s; A) Steatosis: Significantly increased CB_1_ expression in hepatitis C patients with steatosis, B) Steatosis grades: Significantly increased CB_1_ expression with increasing steatosis grade.

There was no difference in CB_1_ expression between genotypes 1 and 3, nor was there any association between CB_1_ and portal inflammatory activity. The presence of steatosis was associated with significantly increased CB_1_ expression in the hepatitis C cohort (figure 5A, *p*<0.05) and CB_1_ expression increased with steatosis grade (figure 5B, p<0.01). Genotype was significantly associated with steatosis grade, so an interaction term was used to test if the association between CB_1_ and steatosis grade was genotype dependent. This demonstrated that CB_1_ expression was highly associated with steatosis grade for genotype 3, but not genotype 1 (*p-*value for interaction term  = 0.006).

We next examined genes that have been shown to be up-regulated by CB_1_ receptor activation and are associated with lipogenesis (table 3). Overall, CB_1_ had a modest correlation with sterol regulatory element binding protein (SREBP-1c; R = 0.21, *p*<0.05) and its downstream target fatty acid synthase (FASN; R = 0.25, *p*<0.05), but this was significantly stronger in genotype 3 patients (SREBP-1c; R = 0.37, FASN; R = 0.39, p<0.05 for both) and not present in those with genotype 1 disease. CB_1_ had a modest correlation with insulin resistance as measured by the HOMA-IR (R = 0.23, *p*<0.05), but had no association with other steatogenic factors such as measures of adiposity, BMI, lipids, or increasing age.

**Table 3:** Rank correlations between CB_1_ and factors associated with steatosis in HCV by genotype.

	SREBP-1c	FASN	HOMA-IR	BMI	HDL	TG	Age
CB_1_ - HCV all	0.21*	0.25*	0.23*	0.10	0.03	0.01	0.15
CB_1_ - HCV G1	0.08	0.19	0.19	0.11	0.11	−0.04	0.21
CB_1_ - HCV G3	0.37*	0.39*	0.24	0.20	0.01	0.02	0.14

*p-value <0.05.

SREBP-1c; Sterol regulatory element binding protein, FASN; fatty acid synthase, HOMA-IR; homeostasis model assessment of insulin resistance, BMI; body mass index, HDL; high density lipoprotein, TG; triglyceride.

### Independent association between CB_1_, steatosis and fibrosis

Multivariate analysis was performed to determine if CB_1_ was independently associated with steatosis and fibrosis in CHC and controls. Input variables included CB_1_, BMI, HOMA-IR, ALT, age, viral load, gender, steatosis grade and fibrosis stage. For fibrosis, input variables identified on univariate analysis were CB_1_, HOMA-IR, BMI, age and steatosis grade. CB_1_ remained a significant independent predictor of increasing fibrosis stage (*p* = 0.04), as did HOMA-R (*p* = 0.008), BMI (*p* = 0.04) and steatosis grade (*p* = 0.001). For steatosis, input variables were CB_1_, HOMA-IR, viral load, genotype and fibrosis stage. CB_1_ remained an independent predictor of increasing steatosis (*p = *0.03) along with viral load (*p* = 0.007) and genotype (*p*<0.001).

## Discussion

In this study we demonstrate the presence of cannabinoid receptor 1 (CB_1_) in the livers of patients with chronic hepatitis C, a finding that has not been previously reported. We found CB_1_ receptor to be expressed in all patients with CHC, with a significant up-regulation compared to control patients. While CB_1_ expression was highest in those with advanced fibrosis, the levels in patients with early hepatitis C (Fibrosis 0-1 and no steatosis) were still 4-fold greater than that of controls. Moreover, there was a strong positive association between CB_1_ expression and HCV viral load. This suggested a direct viral effect, and led us to examine CB_1_ receptor expression in an *in vitro* system in which infectious virus is produced.

The Huh7/JFH-1 system, first described by Wakita *et al* in 2005 [26], uses full genomic RNA from the JFH1 genotype 2a strain of HCV, isolated from a patient with fulminant hepatitis. Once transfected into the human hepatoma cell line Huh7, JFH-1 virus replicates efficiently and virus particles are produced that are infectious in both tissue culture and chimpanzees [26]. In Huh7 cells infected with HCV (JFH-1) we showed that CB_1_ expression was increased over 8-fold compared to control cells. The enrichment of CB_1_ expression in JFH1-infected cells suggests for the first time that CB_1_ receptor is an HCV-inducible gene. Our results using the JFH-1 subgenomic replicon suggest that HCV structural proteins are essential for induction of CB_1_, as there was no increase seen with nonstructural proteins alone. We were unable to demonstrate an effect of core protein genotype on CB_1_ expression using chimeric viruses, which supports our clinical data showing no difference in CB_1_ expression between patients infected with HCV genotypes 1 and 3.

One limitation of this and other liver biopsy-based human studies relates to the cross-sectional nature of the data. Reports of this type can demonstrate significant associations, but inference of cause and effect are always difficult. We used a number of methods to strengthen our finding that CB_1_ was directly induced by hepatitis C. Firstly, we demonstrated an up-regulation of CB_1_ in those with very mild hepatitis C (F0-1 and no steatosis) compared with controls, and an association with viral load, which would not be expected if this was a non-specific effect of fibrosis or inflammation. We then went on to show that CB_1_ expression in comparable patients with mild hepatitis B (F0-1) was significantly lower (almost 3-fold) than those with mild hepatitis C. Finally, we used a cell culture system to demonstrate a direct relationship between CB_1_ expression and hepatitis C viral infection, both in time course and static experiments. Cannabis smoking did not appear to be a confounder as no difference in expression was seen in the small number of cannabis smokers compared to the remaining cohort. While the numbers were too small to determine if cannabis smoking regulates CB_1_ expression, published reports indicate that this is the case only in daily smokers [4], [24] and these patients were excluded from the study. It should be noted that control subjects had significantly higher BMI and HOMA-IR scores than those with hepatitis C. However, since CB_1_ expression has been associated with insulin resistance and obesity, this would if anything lead to an underestimate of the difference in expression. Further work however clearly needs to be done to define the mechanisms by which HCV up-regulates CB_1_ and the specific viral proteins involved.

The pathophysiology of steatosis in CHC is complex. In HCV genotype 1, steatosis is associated with metabolic disturbance, obesity and insulin resistance [5], while steatosis in genotype 3 appears principally to be virally mediated [32]. HCV is known to directly induce steatosis via interactions between core protein [33], [34] and lipogenic regulators such as SREBP-1c [35] and microsomal triglyceride transfer protein (MTP) [36]. In our cohort, hepatic CB_1_ expression correlated with the extent of steatosis and was significantly up-regulated in those with increased steatosis grade, suggesting CB_1_ receptor activation and signalling. This association was highly significant for genotype 3, but not 1. Moreover, in genotype 3 patients, CB_1_ expression correlated strongly with the lipogenic transcription factor SREBP-1c and its downstream target FASN, but in genotype 1 patients there was no correlation. Clinical studies in CHC have hinted at the importance of CB_1_ stimulation to steatosis, with daily cannabis use a risk factor for steatosis severity in over 300 patients with CHC [24]. In experimental work, endocannabinoid stimulation of CB_1_ mediates diet-induced steatosis, since CB_1_ knockout mice fed a high fat diet are resistant to steatosis [18]. Likewise, treatment of wild type mice with a CB_1_ agonist induces *de novo* fatty acid synthesis via increased hepatic expression of SREBP-1c and its downstream targets FASN and acetyl coenzyme-A carboxylase-1 (ACC1) [18]. Finally, the selective deletion of hepatocyte CB_1_ receptors alone is sufficient to prevent diet [37] and alcohol-induced [22] hepatic steatosis [22], [37].

The endocannabinoid system plays an important role in liver fibrosis. In three murine models of chronic liver injury, CB_1_ receptor antagonism by pharmacological or genetic means reduced fibrosis area, TGF-β1 expression and the accumulation of fibrogenic cells. It has also been shown that CB_1_ can mediate liver fibrosis through effects on apoptosis and the growth of hepatic myofibroblasts [19]. Clinical studies first demonstrated the likely importance of this system in patients with CHC, showing daily cannabis smoking to be independently associated with the progression and severity of fibrosis [4]. In our study, CB_1_ was expressed in all patients with CHC and increased with advancing fibrosis, with the highest levels present in those with cirrhosis. We were unable to show any relationship between CB_1_ receptor expression and inflammatory grade, although this does not exclude that the endocannabinoid system via CB_1_ may mediate fibrosis in this way. Rather, it has been suggested that HCV can directly activate and stimulate hepatic stellate cells through its core and non-structural proteins, or via secretions from infected hepatocytes [38], [39]. In this context, activated HSCs not only secrete collagens and cytokines, but also the endocannabinoid 2-AG, which in turn up-regulates and activates hepatocyte CB_1_ [22]. Stimulation of hepatocyte CB_1_ through this pathway or directly by HCV as we demonstrate will serve to amplify the pathways by which liver fibrosis develops in CHC [2], [32]. It is interesting to note from our immunohistochemistry that CB_1_ receptors were up-regulated on hepatic stellate cells in CHC. One could therefore speculate that the direct pro-fibrogenic interactions between HCV and stellate cells demonstrated by Battaler and collegues [38] may in part, be mediated through and exaggerated by the induction of CB_1_.

There has been much recent interest in the use of CB_1_ antagonists to treat both hepatic and metabolic disease and our findings emphasize the likely usefulness of these compounds in patients with hepatitis C. In addition to the amelioration of steatosis and fibrosis, CB_1_ blockade reduces portal pressure and can reverse mesenteric arterial dilation [40], making them useful in end stage liver disease as well. We speculate that CB_1_ antagonism may also have an inhibitory effect on HCV replication. This is prompted both by the significant, genotype independent association we found between CB_1_ expression and viral load, and by reports that blockade of the SREBP and FASN signalling in hepatitis C cell culture models reduces HCV replication [41], [42]. Clearly this needs further study before any conclusions can be drawn. Unfortunately, several CB_1_ receptor antagonists were recently withdrawn from clinical development including Rimonabant (SR141716A) and Taranabant (MK-0364). These withdrawals followed an EU medicines safety edict that Rimonabant be suspended due to excessive psychiatric side effects including depression, anxiety and suicide [43]. Nonetheless, our findings and those of others, showing the importance of hepatic CB_1_ receptors in CHC, suggest that the development of a peripherally selective CB_1_ antagonist with minimal neurotoxicity remains a promising future option.

In conclusion, we have demonstrated that CB_1_ receptor is widely expressed in the livers of patients with CHC and is associated with advanced fibrosis and steatosis. Importantly, CB_1_ is also highly enriched in those with low fibrosis and is induced by HCV in a cell culture system, findings that underscore the unique susceptibility of patients with CHC to cannabis-induced liver damage. CB_1_ has already been implicated in the genesis of fibrosis and steatosis in experimental and metabolic liver disease. We have now shown that it is important in CHC and may be a future target for pharmacotherapy in this disease.

## Supporting Information

Figure S1 Representative immunostaining for CB1 receptor protein in hepatitis C patients with high and low CB1 expression. A) and B) High CB1 expression and advanced fibrosis showing strong, diffuse cytoplasmic and nuclear immunostaining primarily of hepatocytes. C) and D) Low CB1 expression and low fibrosis showing low intensity and patchy cytoplasmic and nuclear immunostaining of hepatocytes.
(6.68 MB TIF)

## References

[1] World Health Organisation Initiative for vaccine research (IVR) Hepatitis C page.. http://www.who.int/vaccine_research/diseases/viral_cancers/en/index2.html.

[2] van der Poorten D, George J (2008). Disease-specific mechanisms of fibrosis: hepatitis C virus and nonalcoholic steatohepatitis.. Clin Liver Dis.

[3] McCaughan GW, George J (2004). Fibrosis progression in chronic hepatitis C virus infection.. Gut.

[4] Hezode C, Roudot-Thoraval F, Nguyen S, Grenard P, Julien B (2005). Daily cannabis smoking as a risk factor for progression of fibrosis in chronic hepatitis C.. Hepatology.

[5] Hui JM, Sud A, Farrell GC, Bandara P, Byth K (2003). Insulin resistance is associated with chronic hepatitis C virus infection and fibrosis progression [corrected].. Gastroenterology.

[6] Hui JM, Kench J, Farrell GC, Lin R, Samarasinghe D (2002). Genotype-specific mechanisms for hepatic steatosis in chronic hepatitis C infection.. J Gastroenterol Hepatol.

[7] Kumar D, Farrell GC, Fung C, George J (2002). Hepatitis C virus genotype 3 is cytopathic to hepatocytes: Reversal of hepatic steatosis after sustained therapeutic response.. Hepatology.

[8] Asselah T, Rubbia-Brandt L, Marcellin P, Negro F (2006). Steatosis in Chronic Hepatitis C: Why does it really matter?. Gut.

[9] Ohata K, Hamasaki K, Toriyama K, Matsumoto K, Saeki A (2003). Hepatic steatosis is a risk factor for hepatocellular carcinoma in patients with chronic hepatitis C virus infection.[see comment].. Cancer.

[10] Su AI, Pezacki JP, Wodicka L, Brideau AD, Supekova L (2002). Genomic analysis of the host response to hepatitis C virus infection.. PNAS.

[11] Smart RG, Ogborne AC (2000). Drug use and drinking among students in 36 countries.. Addict Behav.

[12] Devane WA, Hanus L, Breuer A, Pertwee RG, Stevenson LA (1992). Isolation and structure of a brain constituent that binds to the cannabinoid receptor.. Science.

[13] Pacher P, Batkai S, Kunos G (2006). The endocannabinoid system as an emerging target of pharmacotherapy.. Pharmacol Rev.

[14] Kunos G, Osei-Hyiaman D, Liu J, Godlewski G, Batkai S (2008). Endocannabinoids and the control of energy homeostasis.. J Biol Chem.

[15] Di Marzo V, Goparaju SK, Wang L, Liu J, Batkai S (2001). Leptin-regulated endocannabinoids are involved in maintaining food intake.. Nature.

[16] Ravinet Trillou C, Delgorge C, Menet C, Arnone M, Soubrie P (2004). CB1 cannabinoid receptor knockout in mice leads to leanness, resistance to diet-induced obesity and enhanced leptin sensitivity.. Int J Obes Relat Metab Disord.

[17] Despres JP, Golay A, Sjostrom L, Rimonabant in Obesity-Lipids Study G (2005). Effects of rimonabant on metabolic risk factors in overweight patients with dyslipidemia.[see comment].. New England Journal of Medicine.

[18] Osei-Hyiaman D, DePetrillo M, Pacher P, Liu J, Radaeva S (2005). Endocannabinoid activation at hepatic CB1 receptors stimulates fatty acid synthesis and contributes to diet-induced obesity.[see comment].. Journal of Clinical Investigation.

[19] Teixeira-Clerc F, Julien B, Grenard P, Tran Van Nhieu J, Deveaux V (2006). CB1 cannabinoid receptor antagonism: a new strategy for the treatment of liver fibrosis.. Nat Med.

[20] Julien, Grenard, Teixeira C, Van N, Li (2005). Antifibrogenic role of the cannabinoid receptor CB2 in the liver.. Gastroenterology.

[21] Munoz-Luque J, Ros J, Fernandez-Varo G, Tugues S, Morales-Ruiz M (2008). Regression of fibrosis after chronic stimulation of cannabinoid CB2 receptor in cirrhotic rats.. J Pharmacol Exp Ther.

[22] Jeong WI, Osei-Hyiaman D, Park O, Liu J, Batkai S (2008). Paracrine activation of hepatic CB1 receptors by stellate cell-derived endocannabinoids mediates alcoholic fatty liver.. Cell Metab.

[23] Gary-Bobo M, Elachouri G, Gallas JF, Janiak P, Marini P (2007). Rimonabant reduces obesity-associated hepatic steatosis and features of metabolic syndrome in obese Zucker fa/fa rats.. Hepatology.

[24] Hezode C, Zafrani ES, Roudot-Thoraval F, Costentin C, Hessami A (2008). Daily cannabis use: a novel risk factor of steatosis severity in patients with chronic hepatitis C.. Gastroenterology.

[25] Scheuer PJ (1991). Classification of chronic viral hepatitis: a need for reassessment.. J Hepatol.

[26] Wakita T, Pietschmann T, Kato T, Date T, Miyamoto M (2005). Production of infectious hepatitis C virus in tissue culture from a cloned viral genome.. Nat Med.

[27] Kato T, Date T, Miyamoto M, Furusaka A, Tokushige K (2003). Efficient replication of the genotype 2a hepatitis C virus subgenomic replicon.. Gastroenterology.

[28] Shaw ML, McLauchlan J, Mills PR, Patel AH, McCruden EA (2003). Characterisation of the differences between hepatitis C virus genotype 3 and 1 glycoproteins.. J Med Virol.

[29] Rasband W ImageJ. U.S. National Institutes of Health, Bethesda, Maryland, USA, 1997-2007

[30] Boivin B, Vaniotis G, Allen BG, Hebert TE (2008). G protein-coupled receptors in and on the cell nucleus: a new signaling paradigm?. J Recept Signal Transduct Res.

[31] Ellis J, Pediani JD, Canals M, Milasta S, Milligan G (2006). Orexin-1 receptor-cannabinoid CB1 receptor heterodimerization results in both ligand-dependent and -independent coordinated alterations of receptor localization and function.. J Biol Chem.

[32] Negro F (2006). Mechanisms and significance of liver steatosis in hepatitis C virus infection.. World J Gastroenterol.

[33] Shi ST, Polyak SJ, Tu H, Taylor DR, Gretch DR (2002). Hepatitis C virus NS5A colocalizes with the core protein on lipid droplets and interacts with apolipoproteins.. Virology.

[34] Barba G, Harper F, Harada T, Kohara M, Goulinet S (1997). Hepatitis C virus core protein shows a cytoplasmic localization and associates to cellular lipid storage droplets.. PNAS.

[35] Kim KH, Hong SP, Kim K, Park MJ, Kim KJ (2007). HCV core protein induces hepatic lipid accumulation by activating SREBP1 and PPARgamma.. Biochem Biophys Res Commun.

[36] Perlemuter G, Sabile A, Letteron P, Vona G, Topilco A (2002). Hepatitis C virus core protein inhibits microsomal triglyceride transfer protein activity and very low density lipoprotein secretion: a model of viral-related steatosis.. FASEB Journal.

[37] Osei-Hyiaman D, Liu J, Zhou L, Godlewski G, Harvey-White J (2008). Hepatic CB1 receptor is required for development of diet-induced steatosis, dyslipidemia, and insulin and leptin resistance in mice.. J Clin Invest.

[38] Bataller R, Paik YH, Lindquist JN, Lemasters JJ, Brenner DA (2004). Hepatitis C virus core and nonstructural proteins induce fibrogenic effects in hepatic stellate cells.. Gastroenterology.

[39] Schulze-Krebs A, Preimel D, Popov Y, Bartenschlager R, Lohmann V (2005). Hepatitis C virus-replicating hepatocytes induce fibrogenic activation of hepatic stellate cells.. Gastroenterology.

[40] Parfieniuk A, Flisiak R (2008). Role of cannabinoids in chronic liver diseases.. World J Gastroenterol.

[41] Su AI, Pezacki JP, Wodicka L, Brideau AD, Supekova L (2002). Genomic analysis of the host response to hepatitis C virus infection.. Proc Natl Acad Sci U S A.

[42] Yang W, Hood BL, Chadwick SL, Liu S, Watkins SC (2008). Fatty acid synthase is up-regulated during hepatitis C virus infection and regulates hepatitis C virus entry and production.. Hepatology.

[43] European Medicines Agency (2008). The European Medicines Agency recommends suspension of the marketing authorisation of Acomplia..

