# Supplementary figures and images for: Hepatitis C Virus Induces the Cannabinoid Receptor 1
Source: PLoS One. 2010 Sep 17;5(9):e12841. doi: 10.1371/journal.pone.0012841 (PMC2941472; doi:10.1371/journal.pone.0012841)

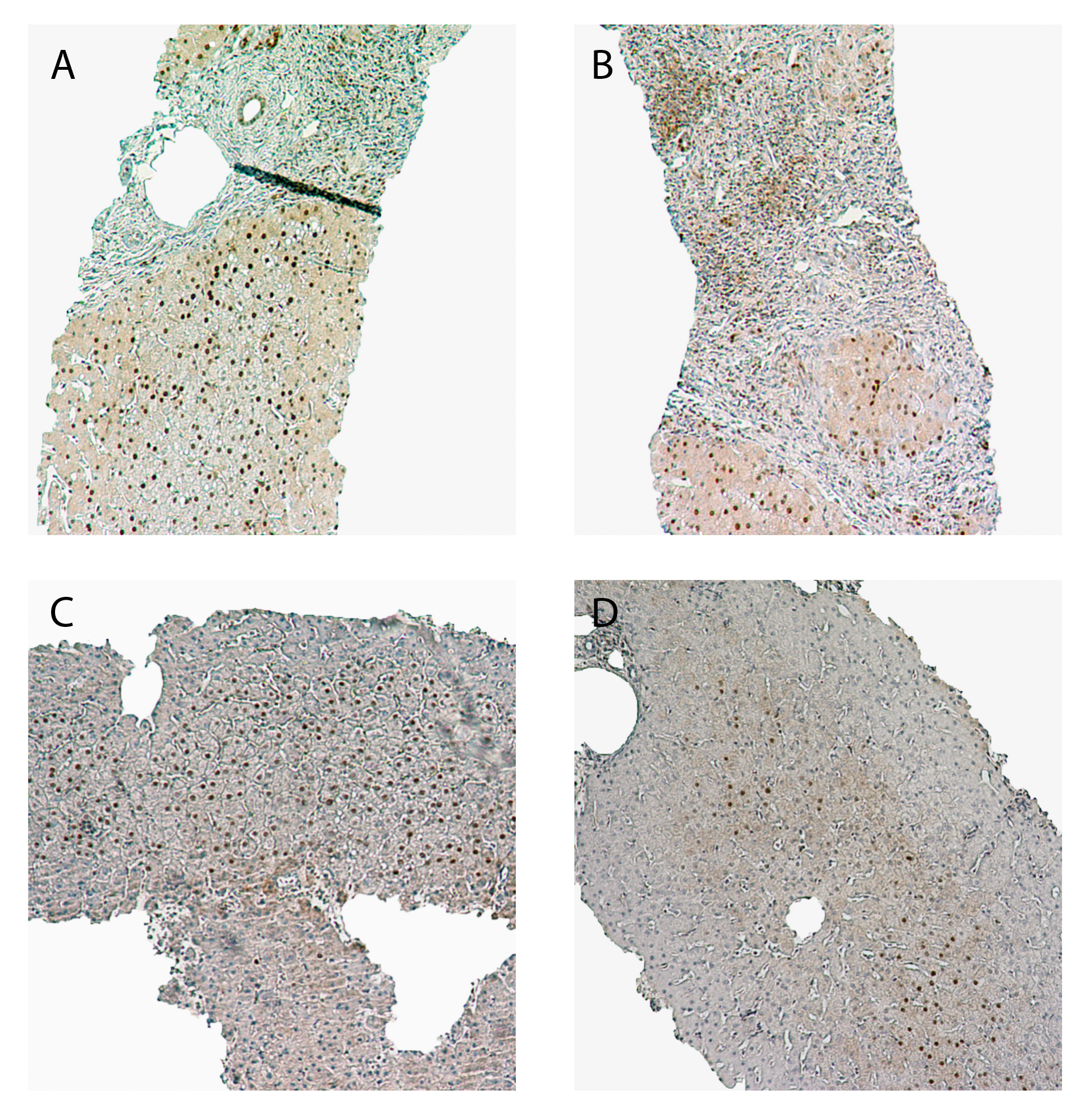

Supplement: Figure S1 — Representative immunostaining for CB1 receptor protein in hepatitis C patients with high and low CB1 expression. A) and B) High CB1 expression and advanced fibrosis showing strong, diffuse cytoplasmic and nuclear immunostaining primarily of hepatocytes. C) and D) Low CB1 expression and low fibrosis showing low intensity and patchy cytoplasmic and nuclear immunostaining of hepatocytes. (6.68 MB TIF) [file pone.0012841.s001.tif]
